# Heteroleptic La^III^ Anilate/Dicarboxylate Based Neutral 3D-Coordination Polymers

**DOI:** 10.3390/molecules26092486

**Published:** 2021-04-24

**Authors:** Olesya Y. Trofimova, Arina V. Maleeva, Irina V. Ershova, Anton V. Cherkasov, Georgy K. Fukin, Rinat R. Aysin, Konstantin A. Kovalenko, Alexandr V. Piskunov

**Affiliations:** 1 G. A. Razuvaev Institute of Organometallic Chemistry of the Russian Academy of Sciences, Tropinin Str., 49, 603137 Nizhny Novgorod, Russia; olesya@iomc.ras.ru (O.Y.T.); arina@iomc.ras.ru (A.V.M.); irina@iomc.ras.ru (I.V.E.); ach@iomc.ras.ru (A.V.C.); gera@iomc.ras.ru (G.K.F.); 2 A. N. Nesmeyanov Institute of Organometallic Chemistry of the Russian Academy of Sciences, Vavilova Str., 28, 119991 Moscow, Russia; aysin.rinat@gmail.com; 3 Nikolaev Institute of Inorganic Chemistry, Siberian Branch of Russian Academy of Sciences, Acad. Lavrentiev Ave., 3, 630090 Novosibirsk, Russia; k.a.kovalenko@niic.nsc.ru

**Keywords:** metal-organic framework, lanthanum, anilate, carboxylate, crystal structure, thermal stability

## Abstract

Three new 3D metal–organic frameworks of lanthanum based on mixed anionic ligands, [(La_2_(pQ)_2_(BDC)_4_)·4DMF]_n_, [(La_2_(pQ)_2_(DHBDC)_4_)·4DMF]_n_, [(La_2_(CA)_2_(BDC)_4_)·4DMF]_n_ (pQ—dianion of 2,5-dihydroxy-3,6-di-tert-butyl-para-quinone, CA—dianion of chloranilic acid, BDC-1,4-benzenedicarboxylate, DHBDC-2,5-dihydroxy-1,4-benzenedicarboxylate and DMF-N,N′-dimethylformamide), were synthesized using solvothermal methodology. Coordination polymers demonstrate the rare **xah** or **4,6T187** topology of a 3D framework. The homoleptic 2D-coordination polymer [(La_2_(pQ)_3_)·4DMF]_n_ was obtained as a by-product in the course of synthetic procedure optimization. The thermal stability, spectral characteristics and porosity of coordination polymers were investigated.

## 1. Introduction

Metal−organic frameworks (MOFs) and coordination polymers (CPs) are an emerging class of microporous solids, which have been intensively studied during the last two decades due to their structural and functional diversity. They have potential applications as gas adsorbents [1,2,3]; luminescent [4,5,6,7]; electrochemical or photophysical sensors [8,9]; and catalytic [10,11,12], optical [13], electrically conductive [14], and magnetic materials [15,16,17,18]. The physical and chemical properties, crystalline structure and topologies of coordination polymers depend on the properties of the organic ligands and/or the metal ions.

The most used types of ligands for MOFs’ construction are various di-, tri-, and tetracarboxylic acids [13]. Their use allows a variety of structures of different dimensions to be obtained, from linear to 3D frames. Among the unique modern trends is the combination of various anionic bridges to obtain heteroleptic polymeric systems. The combination of two or more types of ligands in one link leads to the manifestation of the unusual properties of MOFs and CPs [1,2,4,7]. For example, Fedin and coworkers [1,2] have shown that zinc complexes’ design based on dicarboxylic acids and diatomic alcohols allows the synthesis of functional coordination polymers with outstanding characteristics, excellent adsorption selectivity of benzene over cyclohexane, for example.

Another promising and rapidly developing ligand system in the construction of MOFs is a variety of anilates - derivatives of 2,5-dihydroxy-1,4-benzoquinone [18]. A unique feature of these ligands is their redox activity. Double-deprotonated anilates can exist in five redox states in complexes with metals. The introduction of different substituents in the 3 and 6 positions of the quinoid ring allows for a wide range of variations in their electronic and steric properties. Most commonly, researchers use chlorine [16,19,20], bromine [17,21,22], fluorine [23,24], nitro [25,26] and cyano-substituted [22,27,28] anilate ligands. The first paper on the use of 3,6-di-tert-butyl-2,5-dihydroxy-para-benzoquinone in the construction of 2D layered lanthanum MOFs was published [29]. Lanthanide MOFs/CPs are of great interest for their outstanding optical and magnetic properties [4,6,22,28,30,31,32,33]. They may appear as multifunctional materials for electro-optical, data storage, and sensing applications [5,27,34,35,36]. The first examples of heteroleptic NIR-Emitting Yb^III^/anilate-based coordination polymer nanosheets were prepared recently using terephthalic dicarboxylate coligands [7]. These 2D nanosheets are ideal objects for performing advanced photophysical studies by an innovative multiprobe approach. The research of perturbations of photoluminescence induced by different solvents, both aromatic and aliphatic, bearing electron-withdrawing and electro-donating groups, has demonstrated high-sensitivity to nitrobenzene [7].

In the present work, we report the synthesis and characterization of the first 3D-heteroleptic anilate/carboxylate/La(III) metal organic frameworks. These compound are formulated as [(La_2_(pQ)_2_(BDC)_4_)·4DMF]_n_ (1), [(La_2_(pQ)_2_(DHBDC)_4_)·4DMF]_n_ (2) and [(La_2_(CA)_2_(BDC)_4_)·4DMF]_n_ (3) (pQ—dianionic form of 2,5-dihydroxy-3,6-di-tert-butyl-para-quinone, CA—dianionic form of chloranilic acid, BDC—1,4-benzenedicarboxylate, DHBDC—2,5-dihydroxy-1,4-benzenedicarboxylate, DMF—N,N′-dimethylformamide).

## 2. Results and Discussion

**Scheme 1:**
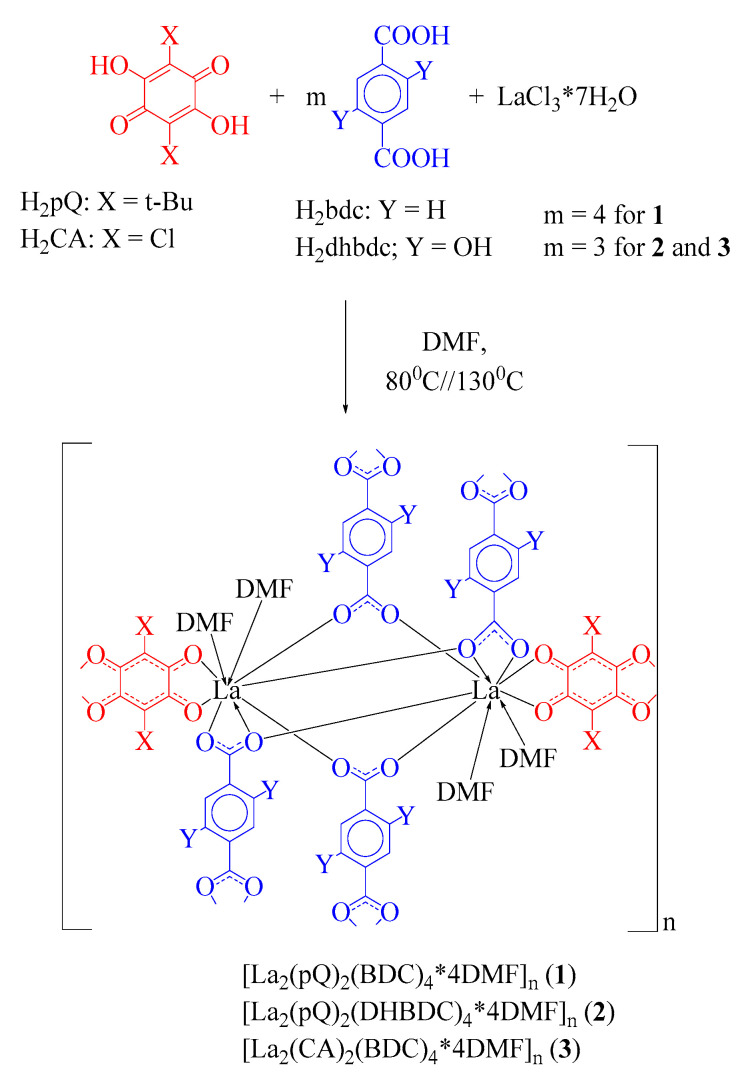
Synthetic scheme for the preparation of coordination polymers **1**–**3**.

Compounds **1**–**3** were synthesized by a solvothermal technique in DMF (Scheme 1).

The synthetic procedure involves consecutive two-stage heating of the reaction mixture at 80 and 130 °C. This ensures a high yield of violet crystals of heteroleptic reaction products. The reaction under conditions of single-stage heating of the components in a DMF solution is accompanied by the formation of crystalline colorless lanthanum carboxylates and red homoleptic anilate derivatives as by-products. Additionally, an increase in the carboxylate ligand’s content in the initial reaction mixture contributes to an increase in the yield of the target product. Therefore, for polymers **1** and **2**, the optimal ratio is H_2_pQ:H_2_bdc (H_2_dhbdc) = 1:4. For the chloranilic acid derivative **3**, the ratio is H_2_CA:H_2_bdc = 1:3. It should be noted that the optimal ratio of reagents differs from the stoichiometric one. This is obviously due to the different rates of formation and crystallization of homoleptic polymers. Additionally, the anilate ligands have a stronger affinity toward the La(III) ion than the carboxylate one. This assertion was forecasted by the authors of [7]. The interaction Yb(III)/anilate/dicarboxylate with stoichiometric (2:1:1) ratio in the reaction produces coordination polymers with the 2:2.5:0.5 or 2:2:1 formula ratios [7]. A significant increase in the content of dicarboxylic acid in the reaction mixture makes it possible to obtain a polymer with a component ratio of La(III)/anilate/dicarboxylate—2:2:4, which dramatically affects the resulting CPs’ structures. In contrast to [7], it is possible to obtain three-dimensional structures instead of layered ones.

**Scheme 2:**
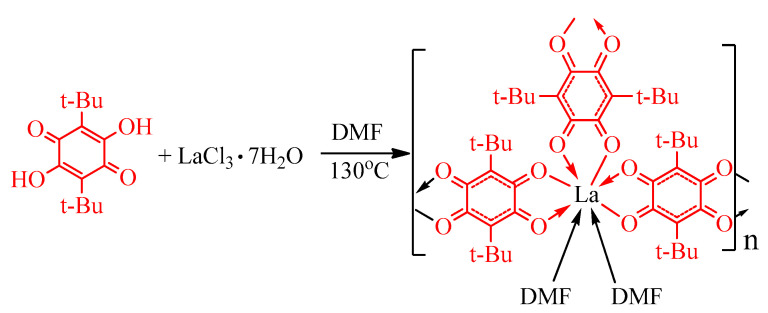
Synthetic scheme for the preparation of coordination polymer **4**.

The La(III) CP (**4**) bearing pQ^2−^ and DMF ligands only was prepared by the test synthesis excluding the dicarboxylate component (Scheme 2).

The SC XRD studies of **1**–**3** compounds were performed, revealing a series of new MOFs with similar structural compositions. MOFs **1** and **2** crystallize in the triclinic *P-1* space group, while **3** crystallizes in the monoclinic *P2_1_/n*. MOFs **1** and **2** display a rarely **xah** [37] topology of the 3D framework. The topology of the underlying network of **3** is **4,6T187** in the standard representation of the valence-bonded MOFs.

**Figure 1:**
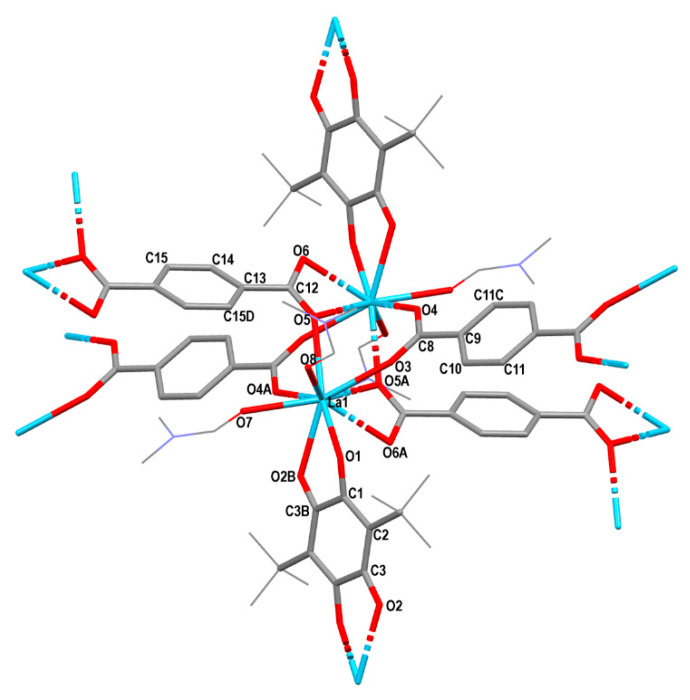
The molecular structure of the binuclear unit in **1**. Color code: La, light blue; C, gray; Cl, green; O, red; N, violet.

**Figure 2:**
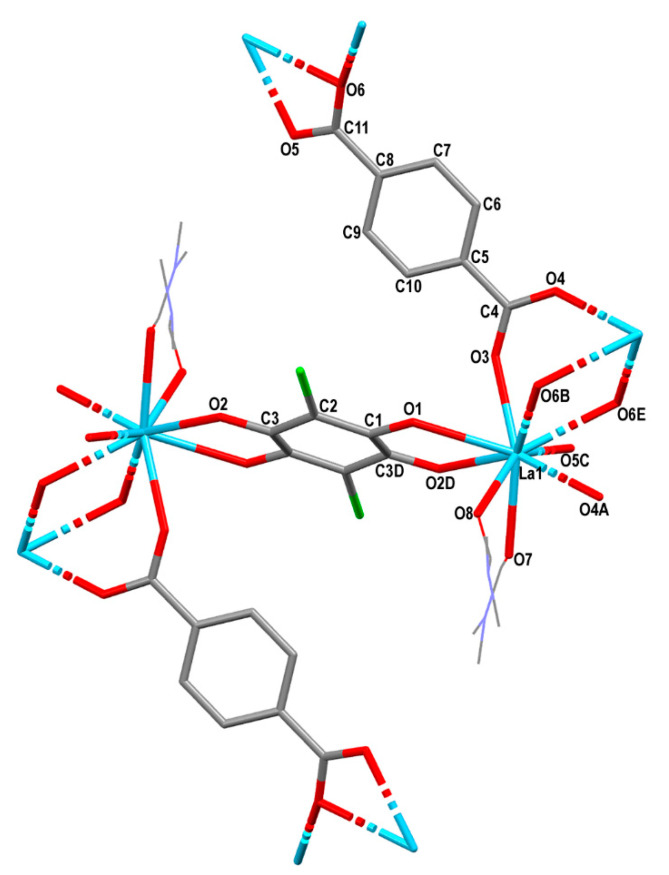
The molecular structure of the crystallographic unit in **3**. Color code: La, light blue; C, gray; Cl, green; O, red; N, violet.

**Figure 3:**
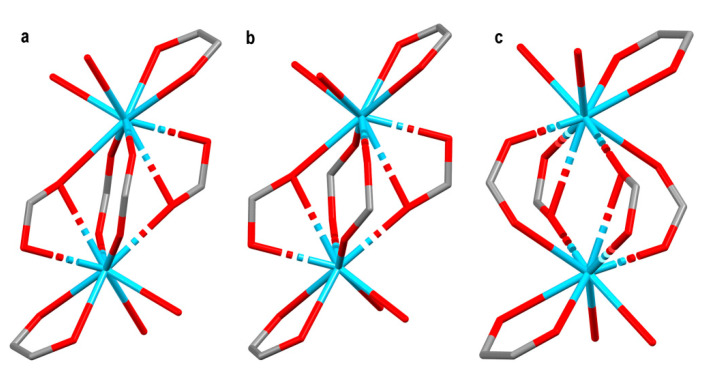
The core of the binuclear units of **1** (**a**), **2** (**b**) and **3** (**c**). Color code: La, light blue; C, gray; O, red.

The simplest unit of **1**–**3** comprises two La(III) centers (Figure 1 and Figure 2). They are bridged by four carboxylate ligands forming the Chinese-lantern structure. Each La(III) center coordinates five oxygen atoms from four BDC^2−^ (**1**, **3**) or DHBDC^2−^ (**2**) anions, two oxygen atoms from pQ^2−^ (**1**, **2**) or CA^2−^ (**3**) anions, and two oxygen atoms of two DMF coordination molecules (Figure 3). Thus, the formal coordination number of La^3+^ in **1**–**3** is nine. The La…La distances in these dimeric units are 4.1978(4) Å in **1**, 4.2239(2) Å in **2** and 4.1725(5) Å in **3**.

**Figure 4:**
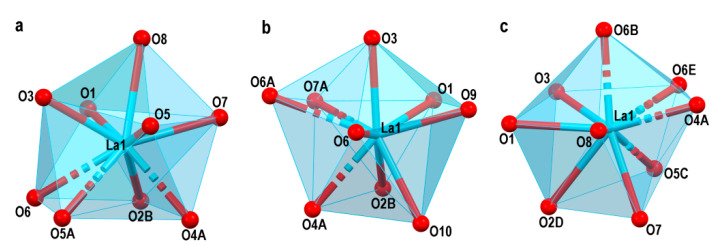
The coordination polyhedron of La cations in **1** (**a**), **2** (**b**) and **3** (**c**).

Systematic analysis of the coordination geometries around the La(III) centers using SHAPE 2.1 software [38,39] reveals that the nine coordinated [40] lanthanide centers of complexes **1–3** adopt different geometries. The coordination polyhedron of lanthanum ion in compound **1** is best described as a spherical capped square antiprism (CSAPR-9, minimum CShM value is 1.383) (Figure 4a). The bottom and top edges are formed by O(2B), O(4A), O(5A), O(6) and O(1), O(3), O(5), O(7) atoms, respectively. The oxygen O(8) of one of the coordinated DMF molecules occupies the capped vertex. Coordination polyhedra in CPs **2** (Figure 4b) and **3** (Figure 4c) are quite close to the muffin (MFF-9, minimum CShM values are 0.926 and 0.868 for **2** and **3**, respectively). The bottom base of these muffins include O(2B), O(4A), O(10) and O(2D), O(5C), O(7) atoms for **2** and **3**, respectively. Apical vertexes depicted by oxygens O(3) (compound **2**) and O(6B) (compound **3**). Pentagonal planes consist of O(1), O(6A), O(7A), O(6), O(9) and O(1), O(3), O(4A), O(6E), O(8) atoms for **2** and **3**, respectively.

**Figure 5:**
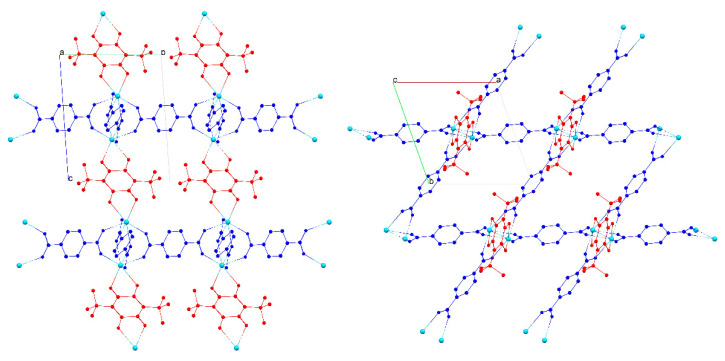
The fragment of crystal packing of **1** along the (*100*) (**left**) and (*001*) (**right**) vectors. DMF molecules are omitted for clarity. Color code: La, light blue; anilic ligands, red; dicarboxylate ligands, blue.

**Figure 6:**
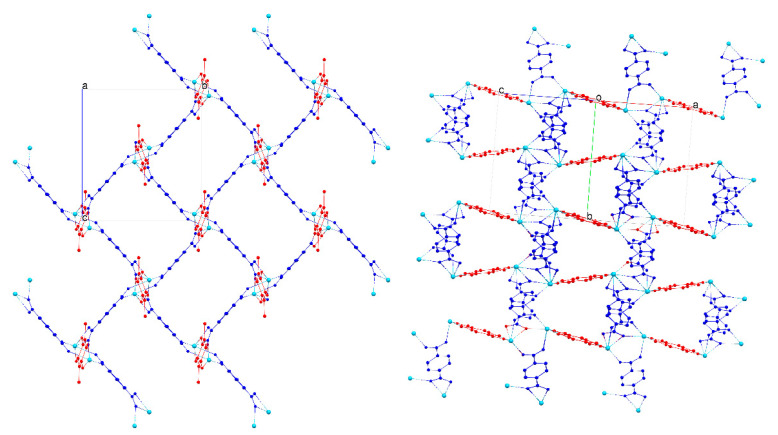
The fragment of crystal packing of **3** along the (*100*) (**left**) and (*101*) (**right**) vectors. DMF molecules are omitted for clarity. Color code: La, light blue; anilic ligands, red; dicarboxylate ligands, blue.

**Figure 7:**
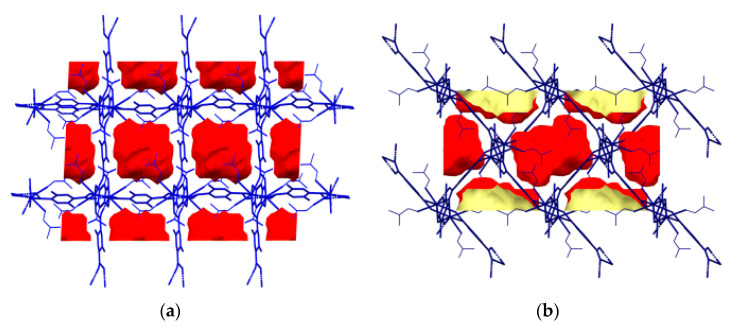
The voids in crystal packing of **2** along the (*001*) vector (**a**) and **3** along the (*100*) vector (**b**). The voids volumes were computed with probe radius 1.2 Å and approx. grid spacing 0.7 Å. Color code: the outer side of the voids, red; the inner side, yellow.

A large excess of dicarboxylic acid contributes to the formation of layers constructed from carboxylate lanterns (Figure 5, Figure 6 and Figure 7). The latter form two-dimensional grids with four-membered cells. The structures of polymers **1** and **2** are close to each other. The carboxylate layers in them are almost flat. In derivative **3**, a zigzag formation of the layers is observed, which leads to its thickening. The layers in **1**–**3** are cross-linked into a three-dimensional structure through anilate ligands. This mechanism of a three-dimensional framework formation explains the difference between the here presented results and those obtained in the preparation of heteroleptic ytterbium complexes [7]. In the case of an excess of the anilic acid, a typical for such complexes (6,3)-2D topology of six-membered rings with hexagonal cavities is formed on the first stage, and dicarboxylate ligands replace some anilate ligands in this lattice. Thus, the predominance of anilic over dicarboxylate ligands in the reaction mixture forms a two-dimensional coordination polymer structure. At the same time, the inverse ratio of the reagents leads to a 3D framework.

**Scheme 3:**
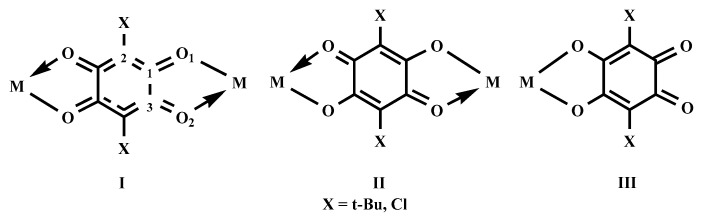
Three different coordination modes of the anilate-type ligand in metal complexes.

The dianionic form of anilate ligands can bind to metals either as a bridging (bis)bidentate ligand, which has a dianion-like structure (Scheme 3, type I and II), or as a terminal bidentate ligand with an ortho-quinoid structure (type III) [41,42]. The dianions of 2,5-dihydroxy-3,6-di-tert-butyl-para-quinone in **1**–**2** and chloranilic acid in **3** consist of two delocalized π-electron systems connected by single C-C bonds (1.552(3) Å). Other C-C distances of six-membered cycle and C-O bonds lies in the narrow ranges 1.404(3)–1.409(3) Å and 1.267(2)–1.271(2) Å, respectively. Such delocalization of electron density in the quinoid ring is characteristic of the bridging mode of anilate derivatives.

**Scheme 4:**
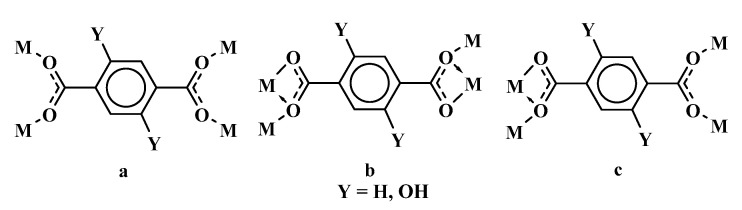
The coordination modes of dicarboxylate ligands (**a**,**b**) in compounds **1** and **2**, and (**c**) in **3**.

Ligands BDC^2−^ in **1** and **3** or DHBDC^2–^ in **2** adopted bridging mode, linking four La^3+^ ions. The types of bridging are shown in Scheme 4. In compounds **1** and **2**, two out of four dicarboxylate ligands act as a linker with each C(O)O-group coordinated by μ_2_-κ^1^: κ^1^-type (type a in Scheme 4), while in the other two ligands, the functional groups have the μ_2_-κ^1^: κ^2^ coordination mode (type b in Scheme 4) (Figure 3a,b). For all four dicarboxylate ligands in **3**, the μ_2_-κ^1^: κ^2^ coordination mode for one C(O)O-group and coordination by μ_2_-κ^1^: κ^1^-type for another one were observed (type c in Scheme 4, Figure 3c). The La-O_dicarb_ bond distances in **1**–**3** are in the range of 2.466(3)–2.849(2) Å, which are similar to those of other related La(III) compounds with dicarboxylate ligands [43,44]. Selected bond lengths are presented in Table 1.

Symmetry transformations used to generate equivalent atoms:

**1:**  *(A)* −x + 1, −y + 1, −z + 1; *(B)* −x + 1, −y + 1, −z; *(C)* −x, −y + 2, −z + 1; *(D)* −x + 2, −y + 1, −z + 1
**2:**  *(A)* −x + 1, −y + 1, −z + 1; *(B)* −x + 1, −y + 1, −z; *(C)* −x + 1, −y, −z + 1; *(D)* −x, −y + 1, −z + 1
**3:**  *(A)* −x + 3, −y, −z + 2; *(B)* −x + 5/2, y + 1/2, −z + 3/2; *(C)* x + 1/2, −y−1/2, z + 1/2; *(D)* −x + 2, −y, −z + 2; *(E)* x + 1/2, −y −1/2, z + 1/2
**4:**  *(A)* −x + 1, −y + 2, −z + 2; *(B)* −x + 1, −y + 1, −z + 2; *(C)* −x, −y + 1, −z + 1.

According to X-ray structural analysis, compounds **2** and **3** contain free accessible voids filled with solvent molecules. The volume of these voids for compound **2** was estimated at 13.8% of unit cell volume (Figure 7a). The voids volume in **3** is about 15.6% (Figure 7b) [45]. Visually, compounds **2** and **3** lost crystallinity within a day, which is associated with the removal of guest DMF molecules from the pores of compounds. The data of elemental and thermogravimetric analyses confirm the absence of guest DMF molecules in the dried samples of **2** and **3**.

**Figure 8:**
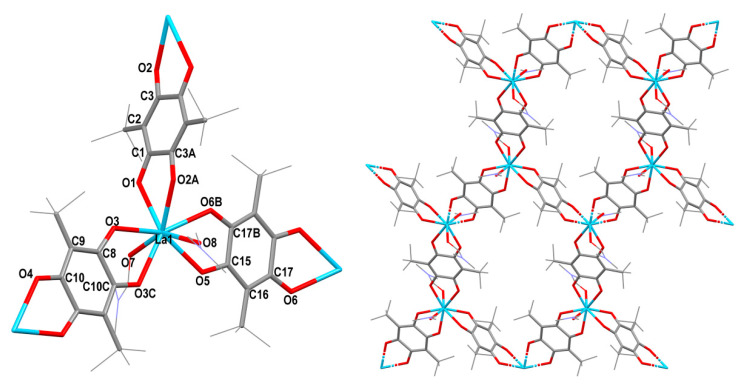
Molecular structure of binuclear unit of **4** (**left**); crystal structure of **4** along the (*100*) vector (**right**). Color code: La, light blue; C, gray; Cl, green; O, red; N, violet.

Metal–organic coordination polymer **4** crystallizes in the triclinic P-1 space group. The topology of the underlying net of **4** is **hcb** and typical for this type of compound [7,21,22,33]. The simplest unit of [(La_2_(pQ)_3_)∙4DMF]_n_ comprises two La(III) cations, three pQ^2−^ dianions and four coordinated N,N′-dimethylformamide ligands (Figure 8). The atom La(III) is 8-coordinated with a distorted triangular dodecahedron geometry (TDD-8, minimum CShM value is 1.148) (Figure 8). The metal–organic coordination polymer **4** has a similar structure to the recently published derivatives [Ln_2_(pQ)_3_∙4DMAA]_n_ (Ln = La, Pr, Nd, DMAA = N,N′-dimethylacetamide) [29]. The main difference is the type of anilate ligands binding to lanthanide ions (Scheme 3). For complexes with dimethylacetamide, two of the three ligands are characterized by type I coordination, and the third one has p-quinoid type II coordination mode. In contrast, all three dianions of 2,5-dihydroxy-3,6-di-tert-butyl-para-quinone in **4** are coordinated by type I and characterized by two delocalized π-electron systems (C-O 1.28(2)-1.32(2) Å) connected by single C–C bonds (1.54(2)-1.551(5) Å). Selected bond lengths in coordination polymer **4** are presented in Table 1.

**Figure 9:**
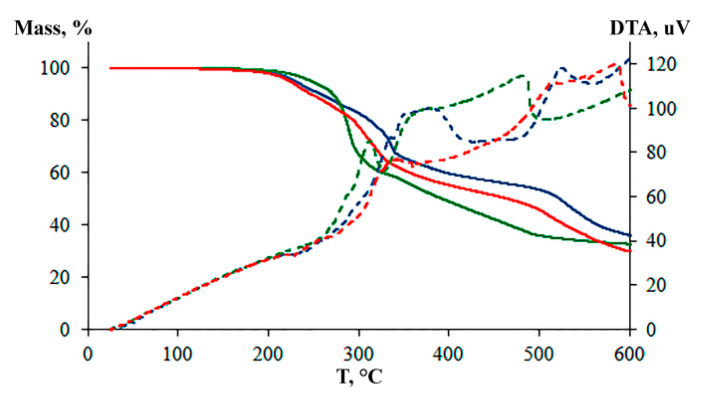
TG curves for **1** (blue line), **2** (green line) and **3** (red line). DTA curves are shown with dash lines.

Thermal stability of obtained compounds **1**–**3** was examined by TGA and DTA (Figure 9). Compounds **1**–**3** started to decompose above 150 °C and showed a slight mass decrease upon heating to 260 °C due to a continuous loss of DMF molecules. The observed mass loss was 10%, 10%, and 8% for **1**, **2**, and **3**, respectively, which corresponds to 1.5 DMF molecules. Up to 150 °C, no weight loss was observed on the TGA curve, which indicates the absence of occluded solvent and guest DMF molecules in the samples. According to DTA data, heating above 270 °C leads to several different processes, which lead to the decomposition of the framework of compounds **1**–**3**. Compound **4** demonstrates thermal stability up to 170 °C. The sample starts to decompose above 170 °C and shows a steady mass decrease upon heating to 250 °C due to a continuous loss of coordination solvent molecules. The observed mass loss equal to 11% corresponds to two DMF molecules in counting on the simplest unit of [(La_2_(pQ)_3_)∙4DMF]. Decomposition of the framework occurred at 298 °C. TG curves for **4** are presented in Figure S2 (ESI).

**Figure 10:**
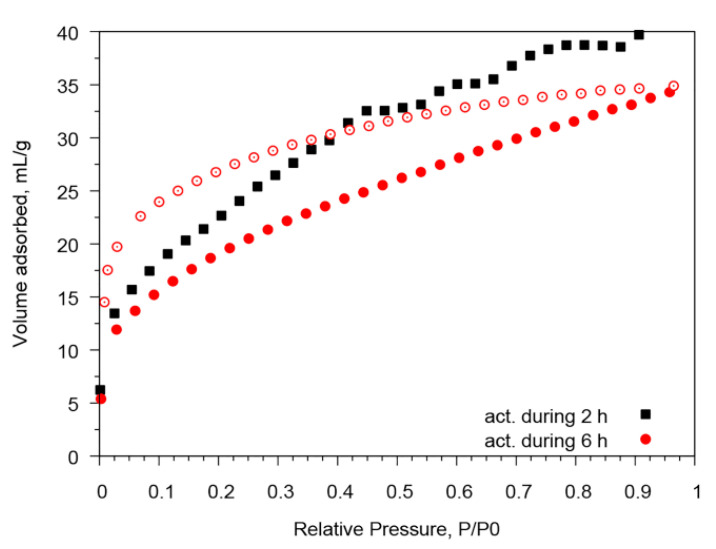
CO_2_ adsorption (•, ▪)-desorption (◦) isotherms at 195 K on **1** activated during 2 and 6 h s.

The porous structure of compounds **1**–**3** was investigated using the example of compound **1**, because this compound retains its crystallinity over a prolonged time period. An analysis of the porous structure was performed by a carbon dioxide adsorption technique at 195 K. Initially, the compound **1** was activated in a dynamic vacuum using the standard “outgas” option of the equipment at 453 K for 2 or 6 h. Measured adsorption isotherms of **1** are represented in Figure 10. Compound **1** has low porosity, pore volume and specific surface area. Longer activation leads to a decrease in the adsorption capacity, as evidenced by a drop in the adsorbed volume of CO_2_. Evidently, this is due to the destruction of the 3D structure when the molecules of the coordinated DMF are eliminated. This is also indicated by a significant deterioration of the powder diffractogram after the completion of the sorption–desorption cycle. Calculated parameters of the porous structure are shown in Table 2. Pore size distributions were calculated using the DFT approach, which shows good agreement between the measured and calculated isotherms and is presented in ESI. Isotherms are typical for microporous materials with slit pores, which was also proven by DFT PSD calculations.

## 3. Materials and Methods

### 3.1. Reagents and Methods

All reactants were purchased from Sigma Aldrich. 2,5-dihydroxy-3,6-di-tert-butyl-p-benzoquinone was synthesized according to the previously reported procedure [46]. All synthetic manipulations were performed under Schlenk line conditions. Solvents were purified by standard methods [47]. Elemental analyses were performed with an Elementar Vario El cube instrument. Electronic absorption (UV-vis) spectra in the range 200–900 nm of nujol mulls were recorded on a Carl Zeiss Jena Specord M400 spectrophotometer. IR-spectra of the studied compounds were recorded on a FSM1201 Fourier-IR spectrometer in a nujol using KBr plates in the range 4000–400 cm^−1^. TGA of compound **1**–**3** was measured on a Shimadzu DTG-60H synchronous thermal analyzer instrument from 30 to 600 °C using an Al pan and heated at a rate of 5 °C/min under an air atmosphere. TGA of compound 4 was measured on a Mettler Toledo TGA/DSC3+ instrument from 30 to 500 °C using an PCA pan and heated at a rate of 5 °C/min under a nitrogen atmosphere.

The X-ray data for **1**–**4** were collected with *Bruker D8 Quest* (**1**–**3**) and *Rigaku OD Xcalibur* (**4**) diffractometers (*Mo_Kα_*-radiation, *ω*-scans technique, *λ* = 0.71073Å, *T* = 100 K) using *APEX3* [48] and *CrysAlis^Pro^* [49] software packages. The structures were solved via an intrinsic phasing algorithm and refined by full-matrix least squares against *F*^2^ using *SHELX* [50,51]. *SADABS* [52] and scaling algorithms implemented in *CrysAlis^Pro^* were used to perform absorption corrections. All non-hydrogen atoms in **1**–**4** were found from Fourier syntheses of electron density and refined anisotropically. All hydrogen atoms were placed in calculated positions and refined isotropically in the “riding” model with *U(H)*_iso_ = 1.2*U*_eq_ of their parent atoms (*U(H)*_iso_ = 1.5*U*_eq_ for methyl groups). The crystal data and structure refinement details for compounds **1**–**4** are presented in Table 3.

One solvate DMF molecule in the asymmetric unit of **2** was modelled by *SQUEEZE/Platon* [53]. Coordination polymer **4** was refined as 2-component non-merohedral twin with a domain ratio 0.65/0.35.

The crystallographic data and structure refinement details are shown in Table S1. CCDC 2,074,328 (**1**), 2,074,329 (**2**), 2,074,330 (**3**) and 2,074,331 (**4**) contain the supplementary crystallographic data for this paper. These data are provided free of charge by The Cambridge Crystallographic Data Centre: ccdc.cam.ac.uk/structures.

An analysis of the porous structure was performed by a carbon dioxide adsorption technique using Quantochrome’s Autosorb iQ at 195 K. Cryostat CryoCooler was used to adjust temperature with 0.05 K accuracy. Carbon dioxide adsorption−desorption isotherms were measured within the range of relative pressures from 10 to 3 till 0.995. The specific surface area was calculated from the data obtained on the basis of the conventional BET, Langmuir and DFT models. Pore size distributions were calculated using the DFT method. The database of the National Institute of Standards and Technology available at http://webbook.nist.gov/chemistry/fluid/ (accessed on 23 April 2021) was used as a source of p−V−T relations at experimental pressures and temperatures.

X-ray powder diffraction measurements were performed on a Shimadzu LabX XRD-6100 X-ray Powder Diffractometer. Storage of crystalline compounds after their separation from the mother liquors led to a partial loss of solvate DMF molecules, which led to the destruction of the crystals studied by X-ray diffraction and the formation of a new crystalline phase. Apparently, the formation of this new phase is reflected in the appearance of extra peaks in the powder diffractogram.

### 3.2. Synthesis

#### 3.2.1. Synthesis of **1**

[(La_2_(pQ)_2_(BDC)_4_)∙4DMF]n (**1**). A mixture of LaCl_3_∙7H_2_O (0.04 mmol), H_2_BDC (0.16 mmol) and H_2_pQ (0.04 mmol) in a mole ratio of 1:4:1 in DMF (2 mL) was heated sequentially at 80 °C for 24 h and 130 °C for 48 h in a sealed glass ampule. The obtained purple crystals were separated by the filtration, washed with DMF (2 × 2 mL) and dried on air. Yield was 90% based on LaCl_3_∙7H_2_O. Elemental analysis calculated for C_42_H_54_N_4_O_16_La_2_: C, 43.92; H, 4.74; N, 4.88. Found: C, 43.45; H, 4.62; N 4.81%. IR (Nujol, KBr, cm^–1^): 1671 w, 1648 w, 1607 w, 1587 w, 1489 w, 1347 w, 1308 w, 1257 m, 1219 m, 1200 m, 1154 m, 1107 w, 1086 s, 1065 m, 1047 s, 1018 m, 972 s, 928 s, 903 m, 887 m, 866 s, 841 w, 810 m, 796 m, 760 w, 750 w, 673 w, 656 m, 511 w, 484 w. Electronic absorption spectrum (Nujol, *λ* (nm)): 336, 382, 508.

#### 3.2.2. Synthesis of **2**

[(La_2_(pQ)_2_(DHBDC)_4_)∙4DMF]n (**2**). A mixture of LaCl_3_∙7H_2_O (0.04 mmol), H_2_DHBDC (0.12 mmol) and H_2_pQ (0.04 mmol) in a mole ratio of 1: 3: 1 in DMF (2 mL) was heated sequentially at 80 °C for 24 h and 130 °C for 48 h in a sealed glass ampule. The obtained purple crystals were separated by the filtration, washed with DMF (2 × 2 mL), and dried on air. Yield was 81% based on LaCl_3_∙7H_2_O. Visually, the compound lost its crystallinity within 1 day. According to the data of X-ray structural analysis, compound 2 contains 2 DMF molecules per simplest unit. According to elemental analysis, after the loss of crystallinity, the compound does not contain guest solvent. Elemental analysis calculated for [C_24_H_34_LaN_3_O_11_]: C, 41.60; H, 4.49; N, 4.62. Found: C, 41.20; H, 4.32; N 4.57%. IR (Nujol, KBr, cm^–1^): 3130 w, 1656 w, 1644 w, 1605 w, 1584 w, 1484 w, 1364 m, 1342 w, 1235 w, 1105 w, 1058 s, 1047 s, 1019 s, 970 s, 925 s, 896 s, 873 m, 862 w, 819 w, 813 w, 794 w, 784 w, 733 w, 673 w, 660 m, 617 s, 607 m, 549 w, 542 w, 509 w, 482 w. Electronic absorption spectrum (Nujol, *λ* (nm)): 350, 390, 520–540.

#### 3.2.3. Synthesis of **3**

[(La_2_(CA)_2_(BDC)_4_)∙4DMF]n (**3**). A mixture of LaCl_3_∙7H2O (0.04 mmol), H_2_BDC (0.12 mmol) and H_2_CA (0.04 mmol) in a mole ratio of 1:3:1 in DMF (2 mL) was heated sequentially at 80 °C for 24 h and 130 °C for 48 h in a sealed glass ampule. The obtained purple crystals were separated by the filtration, washed with DMF (2 × 2 mL) and dried on air. Yield was 81% based on LaCl_3_∙7H_2_O. Visually, the compound lost its crystallinity within 1 day. According to the data of X-ray structural analysis, compound 3 contains 0.6 DMF molecules per simplest unit. According to elemental analysis, after the loss of crystallinity, the compound does not contain guest solvent. Elemental analysis calculated for [C_34_H_36_Cl_4_N_4_O_16_La_2_]: C, 36.94; H, 3.28; N, 5.07. Found: C, 37.16; H, 3.22; N 5.39%. IR (Nujol, KBr, cm^–1^): 1676 w, 1654 w, 1604 w, 1587 w, 1512 w, 1439 w, 1418 w, 1312 m, 1296 m, 1252 s, 1151 m, 1130 s, 1106 w, 1164 s, 1015 m, 993 s, 890 s, 862 s, 841 w, 826 m, 816 m, 785 s, 754 w, 675 w, 595 m, 576 w, 540 s, 510 w. Electronic absorption spectrum (Nujol, *λ* (nm)): 350, 520.

#### 3.2.4. Synthesis of **4**

[(La_2_(pQ)_3_∙4DMF]n. A mixture of solid LaCl_3_·7H_2_O (0.1 mmol) and H_2_pQ (0.1 mmol) was placed in a glass ampoule, and N,N′-dimethylformamide (5 mL) was added. Ampoule was evacuated, sealed and heated at 130 °C for 1 day. The obtained burgundy crystals were separated by the filtration, washed twice by 5 mL of N,N′-dimethylformamide and dried on air. Yield was 90% based on H_2_pQ. C_27_H_41_LaN_2_O_8_. Calculated C, 49.10; H, 6.26; N 4.24%. Found C, 49.51; H, 6.20; N 4.34%. IR (Nujol, KBr) cm^−1^: 1661 w, 1592 m, 1536 w, 1472 w, 1445 w, 1379 w, 1339 w, 1254 m, 1211 m, 1202 m, 1105 w, 1059 m, 1047 w, 1013 s, 964 m, 928 s, 900 w, 865 s, 793 m, 725 s, 673 w, 659 w, 621 m, 480 w. Electronic absorption spectrum (Nujol, *λ* (nm)): 352, 540.

## 4. Conclusions

In summary, we reported the first examples of lanthanide heteroleptic anilate/dicarboxylate three-dimensional metal–organic frameworks constructed from mixed dianionic ligands. The synthesis was carried out under conditions of an excess of dicarboxylic acid. This led to the assembly of two-dimensional layers formed by binuclear lanthanum carboxylate lanterns, spliced into three-dimensional structures by anilate crosslinks. The synthesized structures have low porosity and are thermally stable up to a temperature of 150 °C. We believe that the synthetic approach developed in this work will be particularly useful and can be used for the purposeful construction of functional MOFs based on the entire range of lanthanides.

## Figures and Tables

**Table 1:** Selected bond lengths (Å) in **1**–**4**.

**Bond**	**1**	**Bond**	**2∙2DMF**
La(1)-O(1)	2.477(2)	La(1)-O(1)	2.460(2)
La(1)-O(2B)	2.516(2)	La(1)-O(2B)	2.505(2)
La(1)-O(3)	2.513(2)	La(1)-O(3)	2.495(2)
La(1)-O(4A)	2.510(2)	La(1)-O(4A)	2.48(2)
La(1)-O(5)	2.582(2)	La(1)-O(6)	2.514(2)
La(1)-O(5A)	2.731(2)	La(1)-O(6A)	2.849(2)
La(1)-O(6A)	2.608(2)	La(1)-O(7A)	2.518(5)
La(1)-O(7)	2.568(2)	La(1)-O(9)	2.569(2)
La(1)-O(8)	2.580(5)	La(1)-O(10)	2.496(6)
O(1)-C(1)	1.271(2)	O(1)-C(1)	1.267(2)
O(2)-C(3)	1.267(2)	O(2)-C(3)	1.259(2)
C(1)-C(2)	1.409(3)	C(1)-C(2)	1.402(3)
C(1)-C(3B)	1.552(3)	C(1)-C(3B)	1.540(3)
C(2)-C(3)	1.404(3)	C(2)-C(3)	1.411(3)
O(3)-C(8)	1.262(2)	O(3)-C(8)	1.263(4)
O(4)-C(8)	1.263(2)	O(4)-C(8)	1.319(5)
C(8)-C(9)	1.511(2)	C(8)-C(9)	1.499(4)
C(9)-C(10)	1.388(3)	C(9)-C(10)	1.390(4)
C(9)-C(11C)	1.390(3)	C(9)-C(11C)	1.35(2)
C(10)-C(11)	1.389(3)	C(10)-C(11)	1.393(5)
O(5)-C(12)	1.245(2)	O(6)-C(12)	1.275(3)
O(6)-C(12)	1.255(2)	O(7)-C(12)	1.261(4)
C(12)-C(13)	1.501(3)	C(12)-C(13)	1.486(4)
C(13)-C(15D)	1.396(3)	C(13)-C(14)	1.407(4)
C(13)-C(14)	1.398(3)	C(15)-C(13)	1.391(4)
C(14)-C(15)	1.390(3)	C(15)-C(14D)	1.654(6)
**Bond**	**3∙0.6DMF**	**Bond**	**4**
La(1)-O(1)	2.58(2)	La(1)-O(1)	2.463(7)
La(1)-O(2D)	2.572(7)	La(1)-O(2A)	2.458(7)
La(1)-O(3)	2.474(3)	La(1)-O(3)	2.488(6)
La(1)-O(4A)	2.466(3)	La(1)-O(4C)	2.503(6)
La(1)-O(5C)	2.567(3)	La(1)-O(5)	2.488(6)
La(1)-O(6B)	2.501(3)	La(1)-O(6B)	2.495(6)
La(1)-O(6E)	2.729(3)	La(1)-O(7)	2.52(2)
La(1)-O(7)	2.511(6)	La(1)-O(8)	2.53(2)
La(1)-O(8)	2.494(7)	O(1)-C(1)	1.29(2)
O(1)-C(1)	1.253(6)	O(2)-C(3)	1.28(2)
O(2)-C(3)	1.251(6)	C(1)-C(2)	1.378(7)
C(1)-C(2)	1.398(7)	C(1)-C(3A)	1.54(2)
C(1)-C(3D)	1.58(2)	C(2)-C(3)	1.377(7)
C(2)-C(3)	1.414(7)	O(3)-C(8)	1.288(9)
O(3)-C(4)	1.256(6)	O(4)-C(10)	1.28(2)
O(4)-C(4)	1.260(6)	C(8)-C(9)	1.365(7)
O(5)-C(11)	1.252(6)	C(8)-C(10C)	1.550(5)
O(6)-C(11)	1.261(5)	C(9)-C(10)	1.378(7)
C(4)-C(5)	1.508(6)	O(5)-C(15)	1.32(2)
C(5)-C(6)	1.386(7)	O(6)-C(17)	1.28(2)
C(5)-C(10)	1.391(7)	C(15)-C(16)	1.393(7)
C(6)-C(7)	1.393(7)	C(15)-C(17B)	1.551(5)
C(7)-C(8)	1.392(6)	C(16)-C(17)	1.376(7)
C(8)-C(9)	1.391(7)		
C(9)-C(10)	1.389(7)		
C(8)-C(11)	1.497(6)		

**Table 2:** The parameters of porous structure of compound **1**.

Activation	Specific Surface Area/m^2^·g^−1^			V_pore_/cm^3^·g^−1^		V_ads_(CO_2_) ^a^/cm^3^(STP)·g^−1^
	Langmuir	BET	DFT	Total ^a^	DFT	
2 h	237.3	111.2	61.6	0.0859	0.0604	78.9
6 h	145.3	91.3	86.1	0.0727	0.0440	34.3

^a^ at P/P_0_ = 0.95.

**Table 3:** Crystal data and structure refinement details for **1–4**.

Compound	1	2∙2DMF	3∙0.6DMF	4
Formula	C_42_H_54_La_2_N_4_O_16_	C_48_H_68_La_2_N_6_O_22_	C_35.80_H_40.20_Cl_2_La_2_N_4.60_ O_16.60_	C_54_H_82_La_2_N_4_O_16_
Formula weight	1148.71	1358.90	1149.24	1321.06
Crystal system	Triclinic	Triclinic	Monoclinic	Triclinic
Space group	*P-1*	*P-1*	*P2_1_/n*	*P-1*
*a*, Å	10.1266(10)	10.6159(4)	12.4139(5)	10.471(2)
*b*, Å	10.3128(10)	11.4493(4)	12.9185(5)	13.1311(17)
*c*, Å	12.3495(12)	12.4139(4)	14.7089(6)	13.8254(18)
*α*, deg	81.021(3)	90.5437(13)	90	112.436(12)
*β*, deg	75.024(3)	108.8847(13)	103.7170(11)	92.458(16)
*γ*, deg	68.767(3)	92.3540(13)	90	108.076(16)
*V*, A^3^	1158.5(2)	1426.01(9)	2291.57(16)	1642.0(5)
*Z*	1	1	2	1
*d*_calc_, g/cm^3^	1.646	1.582	1.666	1.336
*θ* range, °	2.48–28.74	2.03–35.63	2.46–30.00	2.98–26.60
Crystal size, mm	0.20 × 0.05 × 0.05	0.38 × 0.33 × 0.08	0.20 × 0.10 × 0.06	0.25 × 0.14 × 0.05
*μ*, mm^−1^	1.892	1.559	2.027	1.344
Reflnscollected/unique	18,483/5986	28,659/13,079	31,260/6682	34,062/20,437
Unique reflns [*I* > 2*σ(I)*]	5683	11,716	5298	10,195
*R*_int_	0.0193	0.0242	0.0538	0.0755
*S*(*F*^2^)	1.057	1.056	1.062	1.048
*R*_1_, *wR*_2_ [*I* > 2*σ(I)*]	0.0198, 0.0464	0.0334, 0.0755	0.0489, 0.1144	0.0731, 0.1726
*R*_1_, *wR*_2_ (all data)	0.0220, 0.0471	0.0399, 0.0780	0.0696, 0.1213	0.1361, 0.1916
Δρ_max_/Δρ_min_, e/Å^3^	1.35/−0.61	1.84/−1.21	2.16/−1.49	1.72/−1.23

## Data Availability

The data presented in this study are available in the article and Supplementary Materials.

## Supplementary Materials

The following are available online, Figure S1: Pore size distribution curves for compounds **1;** Figure S2: Pore size distribution curves for TG curve for **4**; Figure S3: PXRD patterns for **1**–**4**; Figure S4: The molecular structure of the binuclear unit in **2**. Shape analysis for **1**–**3**, Shape analysis for **4**.

## Sample Availability

Samples of compounds **1**–**4** are available from the authors.
