# Supplementary material for: Heteroleptic LaIII Anilate/Dicarboxylate Based Neutral 3D-Coordination Polymers
Source: Molecules. 2021 Apr 24;26(9):2486. doi: 10.3390/molecules26092486 (PMC8123117; doi:10.3390/molecules26092486)
Supplement: Supplementary file 1 [file molecules-26-02486-s001.zip › molecules-1190103-supplementary.pdf]

Supporting Information

# Heteroleptic $La^{III}$ Anilate/Dicarboxylate Based Neutral 3D-Coordination Polymers

*Olesya Yu. Trofimova, Arina V. Maleeva, Irina V. Ershova, Anton V. Cherkasov, Georgy K. Fukin, Rinat R. Aysin, Konstantin A. Kovalenko and Alexandr V. Piskunov*

## Table of Contents

Fig. S1. Pore size distribution curves for compounds **1**. ........................................ 2
Fig. S2. TG curve for **4.** .......................................................................... 2
Fig. S3. PXRD patterns for **1-4**. ................................................................. 3
Fig. S4. The molecular structure of the binuclear unit in 2. .................................. 4
Shape analysis for compounds **1-3**. ............................................................ 5
Shape analysis for compound **4**. ................................................................ 6

## Fig. S1. Pore size distribution curves for compounds **1**.

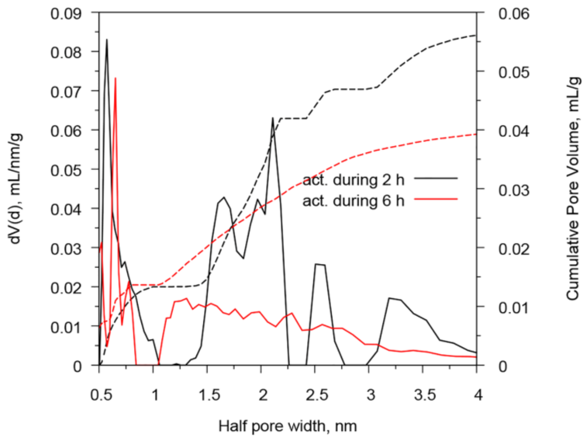


## Fig. S2. TG curve for **4.**

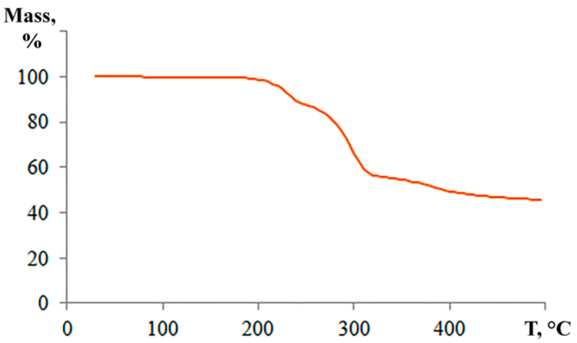

Fig. S3. PXRD patterns for **1-4**.

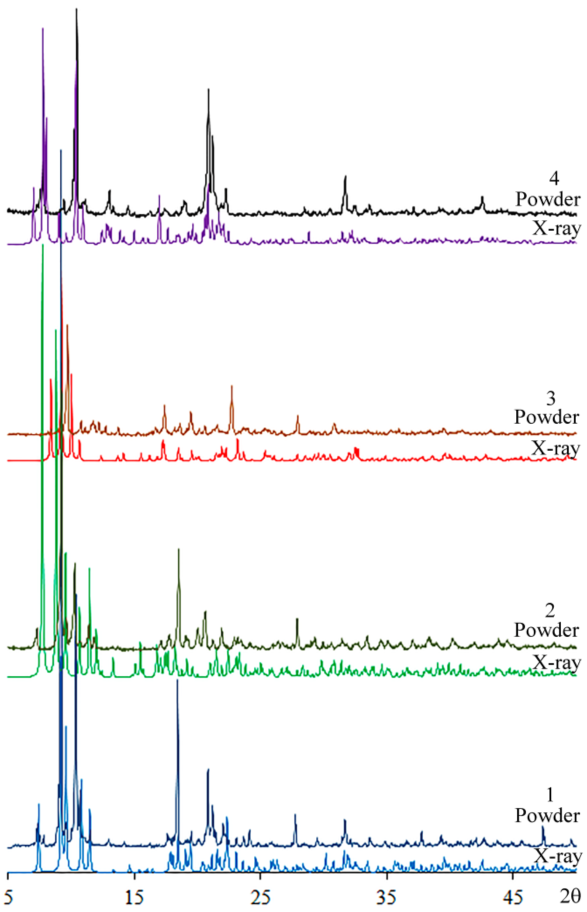

Fig. S4. The molecular structure of the binuclear unit in 2.

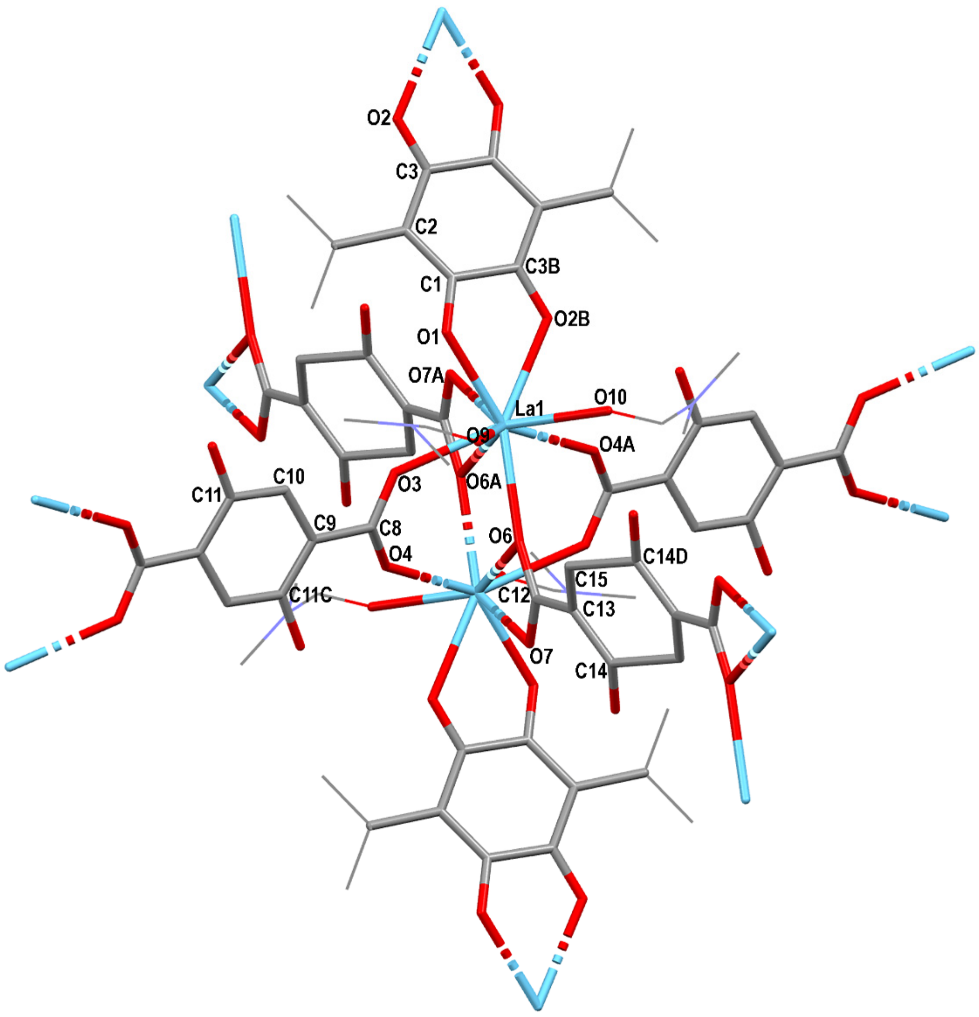

## Shape analysis for compounds **1-3**.

```
-------------------------------------------------------------------------------
S H A P E   v2.1        Continuous Shape Measures calculation
(c) 2013  Electronic Structure Group, Universitat de Barcelona
                Contact:  llunell@ub.edu
-------------------------------------------------------------------------------

DBCLapQ

EP-9                        1 D9h   Enneagon
OPY-9                       2 C8v   Octagonal pyramid
HBPY-9                      3 D7h   Heptagonal bipyramid
JTC-9                       4 C3v   Johnson triangular cupola J3
JCCU-9                      5 C4v   Capped cube J8
CCU-9                       6 C4v   Spherical-relaxed capped cube
JCSAPR-9                    7 C4v   Capped square antiprism J10
CSAPR-9                     8 C4v   Spherical capped square antiprism
JTCTPR-9                    9 D3h   Tricapped trigonal prism J51
TCTPR-9                    10 D3h   Spherical tricapped trigonal prism
JTDIC-9                    11 C3v   Tridiminished icosahedron J63
HH-9                       12 C2v   Hula-hoop
MFF-9                      13 Cs    Muffin
```

| Structure [ML9 ] | EP-9 | OPY-9 | HBPY-9 | JTC-9 | JCCU-9 | CCU-9 | JCSAPR-9 | CSAPR-9 | JTCTPR-9 | TCTPR-9 | JTDIC-9 | HH-9 | MFF-9 |
|---|---|---|---|---|---|---|---|---|---|---|---|---|---|
| compound **1** | 36.538, | 20.629, | 18.934, | 16.676, | 11.332, | 9.185 | 2.553, | 1.384, | 4.001, | 1.711, | 11.116, | 10.683, | 1.500 |
| compound **2** | 34.031, | 22.947, | 18.059, | 15.162, | 10.639, | 10.175, | 2.165, | 1.730, | 2.814, | 2.453, | 13.927, | 10.608, | 0.926 |
| compound **3** | 35.074, | 22.529, | 17.945, | 15.087, | 10.352, | 9.628, | 2.101, | 1.620, | 3.670, | 2.470, | 13.697, | 8.659, | 0.868 |

# Shape analysis for compound **4**.

-------------------------------------------------------------------------------
S H A P E   v2.1        Continuous Shape Measures calculation
(c) 2013  Electronic Structure Group, Universitat de Barcelona
Contact:  llunell@ub.edu
-------------------------------------------------------------------------------

pQ3La2

| | | | |
|---|---|---|---|
| OP-8 | 1 | D8h | Octagon |
| HPY-8 | 2 | C7v | Heptagonal pyramid |
| HBPY-8 | 3 | D6h | Hexagonal bipyramid |
| CU-8 | 4 | Oh | Cube |
| SAPR-8 | 5 | D4d | Square antiprism |
| TDD-8 | 6 | D2d | Triangular dodecahedron |
| JGBF-8 | 7 | D2d | Johnson gyrobifastigium J26 |
| JETBPY-8 | 8 | D3h | Johnson elongated triangular bipyramid J14 |
| JBTPR-8 | 9 | C2v | Biaugmented trigonal prism J50 |
| BTPR-8 | 10 | C2v | Biaugmented trigonal prism |
| JSD-8 | 11 | D2d | Snub diphenoid J84 |
| TT-8 | 12 | Td | Triakis tetrahedron |
| ETBPY-8 | 13 | D3h | Elongated trigonal bipyramid |

| Structure [ML8] | OP-8 | HPY-8 | HBPY-8 | CU-8 | SAPR-8 | TDD-8 | JGBF-8 | JETBPY-8 | JBTPR-8 | BTPR-8 | JSD-8 | TT-8 | ETBPY-8 |
|---|---|---|---|---|---|---|---|---|---|---|---|---|---|
| **compound 4** | 33.495, | 22.913, | 15.109, | 12.177, | 3.465, | 1.148, | 13.268, | 29.184, | 3.397, | 2.575, | 3.490, | 12.999, | 23.339 |
